# Impact of Misclassification Rates on Compression Efficiency of Red Blood Cell Images of Malaria Infection Using Deep Learning

**DOI:** 10.3390/e21111062

**Published:** 2019-10-30

**Authors:** Yuhang Dong, W. David Pan, Dongsheng Wu

**Affiliations:** 1 Department of Electrical and Computer Engineering, University of Alabama in Huntsville, Huntsville, AL 35899, USA; yd0009@uah.edu; 2 Department of Mathematical Sciences, University of Alabama in Huntsville, Huntsville, AL 35899, USA; dw0001@uah.edu

**Keywords:** lossless compression, pattern classification, machine learning, malaria infection, entropy, Golomb–Rice codes

## Abstract

Malaria is a severe public health problem worldwide, with some developing countries being most affected. Reliable remote diagnosis of malaria infection will benefit from efficient compression of high-resolution microscopic images. This paper addresses a lossless compression of malaria-infected red blood cell images using deep learning. Specifically, we investigate a practical approach where images are first classified before being compressed using stacked autoencoders. We provide probabilistic analysis on the impact of misclassification rates on compression performance in terms of the information-theoretic measure of entropy. We then use malaria infection image datasets to evaluate the relations between misclassification rates and actually obtainable compressed bit rates using Golomb–Rice codes. Simulation results show that the joint pattern classification/compression method provides more efficient compression than several mainstream lossless compression techniques, such as JPEG2000, JPEG-LS, CALIC, and WebP, by exploiting common features extracted by deep learning on large datasets. This study provides new insight into the interplay between classification accuracy and compression bitrates. The proposed compression method can find useful telemedicine applications where efficient storage and rapid transfer of large image datasets is desirable.

## 1. Introduction

Malaria occurs in nearly 100 countries worldwide, imposing a huge toll on human health and heavy socioeconomic burdens on developing countries [1]. The agents of malaria are mosquito-transmitted *Plasmodium* parasites. Microscopy is the gold standard for diagnosis; however, manual blood smear evaluation depends on time-consuming, error-prone, and repetitive processes requiring skilled personnel [2]. Ongoing research has therefore focused on computer-assisted Plasmodium characterization and classification from digitized blood smear images [3,4,5,6,7]. Traditional algorithms labeled images using manually designed feature extraction, with drawbacks in both time-to-solution and accuracy [4]. Newly proposed methods aim to apply automated learning to large-size wholeslide images. Leveraging high-performance computing, deep machine learning algorithms could potentially drive true artificial intelligence in malaria research. Concurrently, the convergence of mobile computing, the Internet, and biomedical instrumentation now allows the worldwide transfer of biomedical images for telemedicine applications. Consultation or screening by specialists located in geographically different locations is now possible.

Among recent works on computer-aided diagnosis of malaria infection, two types of images have found prevalent use: light microscopic images and wholeslide images. Recent advances in computing power, improved cloud based services and robust algorithms have enabled the widespread use of wholeslide images [8,9,10,11,12,13]. Higher resolutions can help identify the specific species and the degree of infection. Most of the prior studies utilize light microscopic images [14,15,16,17,18,19,20,21,22,23,24]. While machine learning algorithms have been applied to light microscopic images with relatively low-resolution image processing, higher resolutions would be necessary to identify the specific species and the degree of infection [25].

A notable challenge in such applications is the storage and rapid transfer of massive wholeslide image datasets. Efficient lossless compression methods will be much sought after for malaria infection images. Lossless compression for images has the obvious advantage of suffering no quality loss over lossy methods. Traditional image compression methods seek to minimize the correlation inside the image. For large image datasets, especially medical images that share lots of commonality, the inter-image correlation should also be taken into consideration. Deep learning based neural networks can be trained on samples within the same class to learn the common features shared by these samples. In our prior work [26], we proposed a coding scheme for red blood cell images by using stacked autoencoders, where the reconstruction residues were entropy-coded to achieve lossless compression. Specifically, we trained two separate stacked autoencoders to automatically learn the discriminating features from input images of infected and non-infected cells. Subsequently, the residues of these two classes of images were coded by two independent Golomb–Rice encoders. Simulation results showed that this deep learning approach can provide more efficient compression than several state-of-the-art methods. However, this work assumes that the class labels for the input images are known in advance with perfect classification, which is typically not the case in practice. Hence in this paper, we introduce a more realistic framework where the input images are first classified before being compressed using autoencoders. We study how the accuracy of the classifiers would affect the overall compression ratios for two-class image dataset compression. Note that for traditional lossless compression methods, misclassified samples were not a problem since images were compressed individually. But for compressors based on deep learning methods such as stacked autoencoders, misclassified images fed into autoencoders trained for the other class can lead to very large residues, which could degrade the compression performance. For a more in-depth study, we conduct theoretical analysis based on probabilistic distributions of the prediction residues, and derive formulas for compressed bit rates as a function of classification accuracies. We then use synthesized data based on the models to verify the theoretical results. Next, we use real malaria infection image datasets to evaluate the relations between classification accuracies and compressed bit rates.

In the following, we provide a literature survey on the existing work on joint data compression and classification. While most work in the literature studies data compression and pattern classification separately, some papers [27,28,29] address joint compression and classification, albeit without an in-depth treatment of the interplay between classification and compression. An algorithm on discrete cosine transform (DCT)-based classification scheme was presented in [27] for fractal based image compression, where three classes of image blocks were defined: smooth class, diagonal/sub-diagonal edge class and horizontal/vertical edge class. Two lowest horizontal and vertical DCT coefficients of the given block were used for classification. This reduces the searching space, therefore accelerating the fast fractal encoding process. The author assumed that the classifier was perfect, so no discussion about how the classification accuracy would affect the algorithm was given. A lifting based system was proposed in [28] for Joint Photographic Experts Group (JPEG) 2000 compression to control the trade-off between compression and classification performance. While the paper claims that good classification performance was typically obtained at the expense of some compression performance degradation, no detailed analysis of the interplay between classification and compression was provided. Both [29] and [30] worked on electrocardiogram (ECG) system. A quad-level vector (QLV) was proposed in [29] to support both classification flow and compression flow, in order to achieve better performance with low computational complexity. Wavelet-based features were used in [30] for classification with Support Vector Machine (SVM), where wavelet transform and run length coding were used for compression. Neither of these two papers mentioned the interaction between classification flow and compression flow. Furthermore, several papers [31,32,33] address classification of hyperspectral images (HSI) or multispectral image (MSI) in order to improve the compression performance. Several classification trees were constructed in [31] to study the relationship between compression rate and classification accuracy for lossy compression on HSI. The results showed that high compression rates could be achieved without degrading classification accuracy too much. HSI were also used in [32], where several lossy compression methods were compared on how they would impact classification using pixel-based support vector machine (SVM). Compression of MSI was achieved in [33] by segmentation of image into regions of homogeneous land covers. The classification was conducted via tree-structured vector quantization, and residues were coded using transform coding techniques. The method proposed in [34] is similar to that in [32]. Pixel classification and sorting scheme in wavelet domain was used for image compression. Pixels were classified into several quantized contexts, so as to exploit the intra-band correlation in wavelet domain. Compression and classification of images were combined in [35]. The compressed image incorporated implicit classification information, which can be used directly for low-level classification. Some other researchers [36,37,38] worked with vector quantizer based classifiers to improve compression performance. On the other hand, researchers use neural network [39,40,41,42] for joint classification/compression. A classifier based on wavelet and Fourier descriptor features was employed in [39] to promote lossless image compression. The neural network in [40] was accelerated by compressing image data with an algorithm based on the discrete cosine transform. Singular Value Decomposition (SVD) was used in [41] as compression method that can reduce the size of fingerprint images, while improving the classification accuracy. Two unsupervised data reduction techniques, Autoencoder and self-organizing maps, were compared in [42] to identify malaria from blood smear images.

To the best of our knowledge, there is no in-depth study on the interplay between misclassification rate and compression ratio for lossless image compression methods, in particular, for compression methods based on deep-learning based pattern classification. In this work, to achieve efficient compression of red blood cell images, we use autoencoders to learn the correlations of the image pixels, as well as the correlations among similar images. We train separate autoencoders for images belonging to different classes. Autoencoders can automatically generate hierarchical feature vectors, which reflect common features shared by the images from the same class. We can then recover the original images from the feature vectors. By coding the residues, we can achieve lossless compression on the images. We study how misclassification rate affects the overall compression efficiency.

## 2. Materials and Methods

### 2.1. Construction of the Dataset of Malaria-Infected Red Blood Cell Images

**Figure 1:**
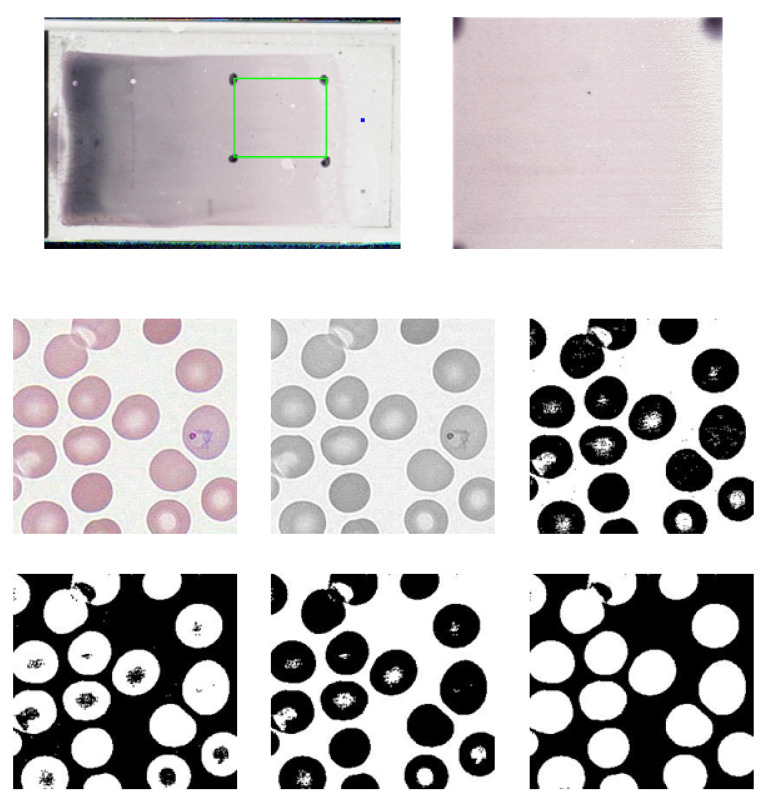
Wholeslide image of malaria-infected RBCs and normal cells. The top left image is the original glass slide after staining. The rectangle delineated in green was cropped out to be the image on the right. After zooming in the area with 100× magnification, we can see the normal cells and infected cells (with the parasites in the ring form) in the leftmost image in the second row. The remaining five grayscale images are the result of step-by-step processing of the leftmost image in the second row. First, the color image is converted into a grayscale image. Then a thresholding operation removes irrelevant info and converts the image into a binary image. The next two steps fills the isolated pixels in both foreground and background. After filling all the holes, we finally got the binary mask. Applying the mask onto the color image, we can extract each single cell image as shown in Figure 2.

**Figure 2:**
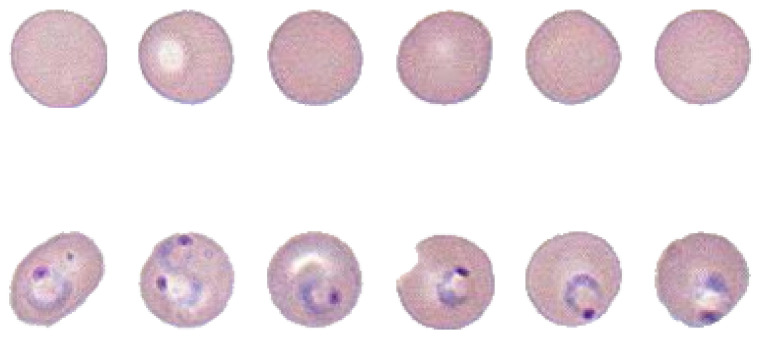
Some example segmented RBC images. (Upper row) normal cells and (lower row) infected cells.

As the result of collaborative research with a group of pathologists from the Medical School of the University of Alabama at Birmingham, we built a dataset of red blood cell (RBC) images extracted from a wholeslide image (WSI) with 100× magnification [43]. The images belong to either one of the following two classes: malaria infected cells and normal cells. Figure 1 shows the glass slide of thin blood smear and the scanned WSI under its highest resolution. The WSI was divided into more than 80,000 image tiles, each with 284 × 284 pixels. Image morphological transforms were applied onto each tile to separate cell samples from the background, as shown in Figure 1 [44]. Some overlapped cells can be separated using Hough circle transform [45]. Finally, all samples were resized into 50 × 50 images, with some examples shown in Figure 2. The entire dataset can be found on our website [46]. For simplicity, we only used red channel for training neural network.

### 2.2. Lossless Compression Using Autoencoders

**Figure 3:**
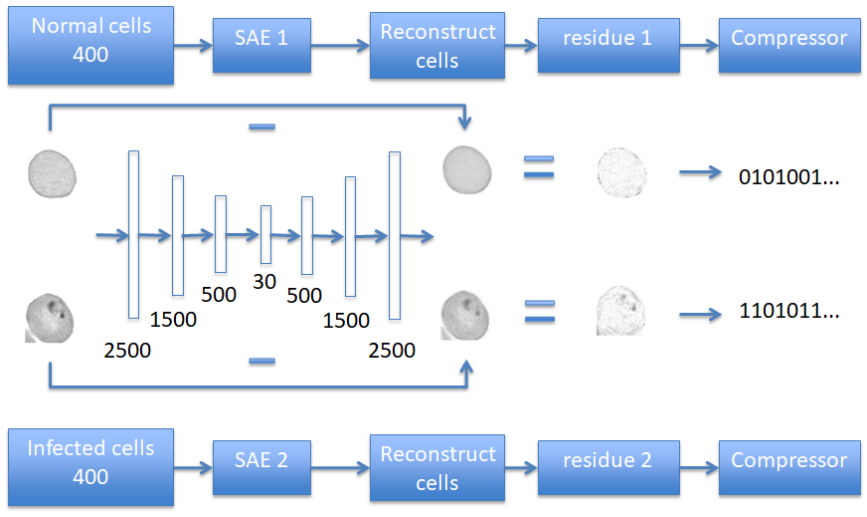
Using autoencoder to compress a 50 × 50 image to a 30-point vector, together with the residue. The residue will be coded using Golomb–Rice Code.

An autoencoder is an artificial neural network that performs unsupervised learning [47], which consists of an encoder and a decoder. The encoder converts the high dimensional input data into a low dimensional feature vector. By reversing this process, the decoder attemps to recover the original data, typically with loss. Back propagation is used when traing the autoencoder to minimize the loss. A more complicated network can be built by stacking several autoencoders together, which will generate a more hierarchical representations of the input data. A fine-tuned autoencoder is able to perform data dimensionality reduction, while extracting features shared by the input data. Thus autoencoders can be used for lossless compression, if the differences between the input data and the reconstructed version are retained and coded efficiently. The flow chart of using stacked autoencoders (SAE) on malaria-infected RBC images is shown in Figure 3.

Two separate stacked autoencoders (SAE) were assigned to images belonging to normal and infected cell classes, respectively, each with 400 samples. Since cell images in the same class share more common features, higher compression efficiency can be acquired than using one SAE for all samples. Each SAE consists of an encoder and a decoder. A cell image of 50×50 was reshaped into a vector of 2500 points, and then fed into encoder. The encoder consists of four layers: The input layer takes in 2500-point vectors, which are reduced by the remaining encoder layers to 1500, 500 and 30 points respectively. Therefore, the stacked autoencoder reduces the input vector into a very low-dimension vector of only 30 entries. Then the decoder attempts to reconstruct the original image from the 30-point vector. The training of the entire autoencoder takes many iterations in order to reduce the difference between the reconstructed image and the original image to a very small value. The resulting residues, along with the 30-point vector are coded to ensure the compression is lossless. Specifically, the residues are compressed efficiently using the Golomb–Rice Code [48].

Unlike most conventional lossless image compression methods such as JPEG2000 [49], which exploits correlations within a single images to be compressed, the autoencoder based method is able to extract common features among a group of similar images. This will allow for potentially more efficient compression on these similarly looking images in a dataset.

### 2.3. Golomb–Rice Coding

If the autoencoder is well trained on the input dataset, the differences (residues) between the reconstructed images and original images tend to center around zero. If the residues are converted to non-negative integers using the following equation:

$$Output=\left\{\begin{array}{cc} -2\cdot Input-1, & \mathrm{if}\;Input<0;\\ 2\cdot Input, & \mathrm{otherwise}, \end{array}\right.$$

then the resulting non-negative values *n* can be approximated by the geometrical distribution with the following probability mass function parameterized by *p*:

(1) $$\begin{array}{c} \mathrm{Prob}\left(n\right)=p^{n}\left(1-p\right), \end{array}$$

where *p* is a real number within the range of (0,1). Golomb–Rice codes are optimal to compress the geometrically distributed source with $p^{m}=\frac{1}{2}$, where *m* is a coding parameter.

The entropy H(p), and expected value E[n] of *n*’s are given below.

(2) $$\begin{array}{cc} & H\left(p\right)=\frac{-\left(1-p\right)\cdot \log_{2}\left(1-p\right)-p\cdot \log_{2}p}{p}, \end{array}$$

(3) $$\begin{array}{cc} & E\left[n\right]=\sum_{n=0}^{\infty }np^{n}\left(1-p\right)=\frac{p}{1-p}. \end{array}$$

Using Equation (3), the parameter *p* can be estimated from the sample mean as follows:

(4) $$\begin{array}{c} p\approx \frac{E(n)}{1+E(n)}. \end{array}$$

The Golomb–Rice coding procedure can be summarized by the following steps:

1. Each non-negative integer *n* to be coded is decomposed into two numbers, *q* and *r*, where n=mq+r, *q* is the quotient of (n/m), and *r* is the remainder.
2. Unary-coding *q* by generating *q* “1”s, followed by a “0”.
3. Coding of *r* depends on if *m* is a power of two:
  - If $m=2^{s}$, *r* can be simply represented using an *s*-bit binary code.
  - If *m* is not power of two, the following thresholds should be calculated first:
    (5) $$\begin{array}{c} A=\left\lceil \log_{2}m\right\rceil ,\mathrm{and}\;B=\left\lfloor \log_{2}m\right\rfloor . \end{array}$$
    If $0\leq r\leq (2^{A}-m-1)$, then *r* is represented by a *B*-bit binary code; Otherwise, if $\left(2^{A}-m\right)\leq r\leq \left(m-1\right)$, then $\left[r+\left(2^{A}-m\right)\right]$ is represented by a *A*-bit binary code.

If $m=2^{s}$, then *s* can be estimated from the sample mean of the input data as

(6) $$\begin{array}{c} s\approx max\left\{0,\left\lceil \log_{2}\frac{E(n)}{2}\right\rceil \right\}, \end{array}$$

and the average codeword length (ACWL) of the Golomb–Rice codes is:

(7) $$\begin{array}{cc} & ACWL=E[q]+1+s, \end{array}$$

where E[q] is the expected value of the quotients *q*.

### 2.4. Joint Classification and Compression Framework

**Figure 4:**
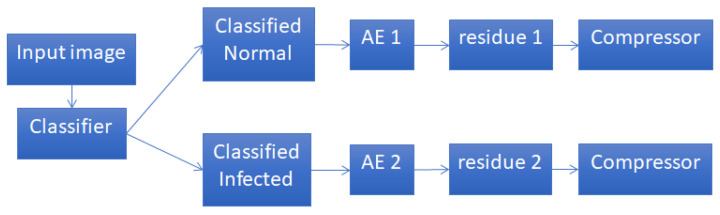
A more realistic framework taking into account misclassification of input images.

Previously, we used autoencoders to exploit the correlations of similar images to achieve high compression on red blood cell images [26]. For this sake, two separate autoencoders were trained using images known in advance to belong to one of the two classes (either normal cells, or malaria infected cells). However, the compression performance suffers if the images fed to the autoencoders actually come from different classes, which is typically the case, where classifiers are not perfect. Therefore, in this work, we study a more realistic framework, as shown in Figure 4, where the input images are first classified before being compressed using autoencoders. So after classification, each class may have some samples that are incorrectly classified. In the following, we conduct an analysis on how the accuracy of the classifiers would affect the overall compression ratios.

### 2.5. Theoretical Analysis

**Figure 5:**
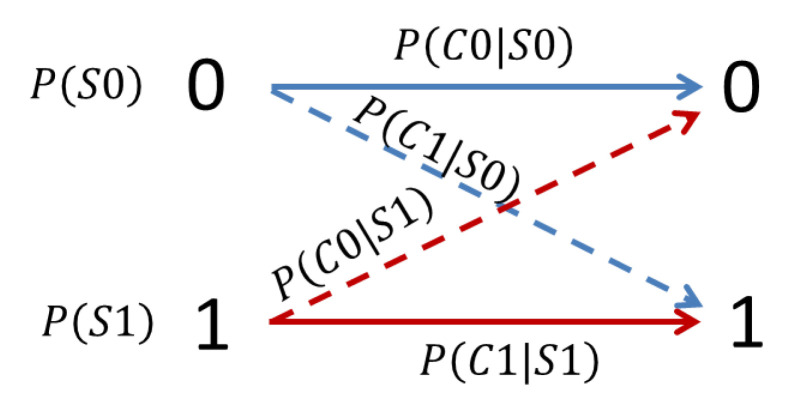
A binary state transition model for cell image classifications. The symbols “1” and “0” to the left represent input source images belonging to either one of two possible classes (infected and normal cells, respectively). The symbols “1” and “0” to the right represent the type of the images an input image is classified into. Arrows represent transitions, e.g., the transition from “1” to “1” means an infected cell is correctly classified. In contrast, the transition from “1” to “0” means an infected cell is incorrectly classified as a normal cell, where the misclassification rate can be described by the conditional probability P(C0|S1) for each class. See Table 1 for the meanings of other probabilities involved.

We employ a binary channel model as illustrated in Figure 5 to characterize the four possible cases of cell image classification, with the meanings of the symbols explained in Table 1. Since there are only two possible classes of input images, we have the source probabilities summing up to unity:

(8) $$\begin{array}{c} P(S0)+P(S1)=1. \end{array}$$

Similarly, the misclassification rates (P(C1|S0) and P(C0|S1)) are related to correct classification rates as:

(9) $$\begin{array}{c} P(C1|S0)+P(C0|S0)=1, \end{array}$$

(10) $$\begin{array}{c} P(C1|S1)+P(C0|S1)=1. \end{array}$$

The source probabilities and the conditional probabilities can be estimated from the image datasets and the pattern classifiers used. We can then derive the joint probabilities of the four possible cases of image classification as listed in Table 1. For example, the joint probability of a cell being normal and correctly classified can be calculated as

(11) $$\begin{array}{c} P(S0,C0)=P(C0|S0)\cdot P(S0). \end{array}$$

**Figure 6:**
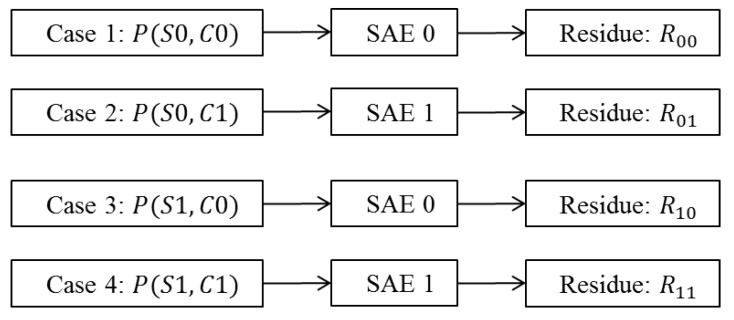
Image compression using stacked autoencoders (SAEs) after pattern classification. “SAE0” and “SAE1” stand for stacked autoencoders trained for normal and infected cells, respectively. $R_{ij}$, where i,j=0,1, denotes the probability distributions of the residues to be entropy coded using Golomb–Rice codes.

Following the joint image classification/compression framework in Figure 4, subsequent to image classification, we use stacked autoencoders to generate residues. As shown in Figure 6, corresponding to different cases of image classifications $\left(S_{i},C_{j}\right)$, we can distinguish four distinct probabilistic distributions of residues $R_{ij}$. where i,j=0,1.

Given that the input images are either for normal cells or infected cells, the following two conditional entropies, H0 and H1, can provide estimates of the compressed bitrates. Specifically,

(12)H0=P(C0|S0)H(R00)+P(C1|S0)H(R01)(13)=[1−P(C1|S0)]H(R00)+P(C1|S0)H(R01),

which is a function of the misclassification rate P(C1|S0). Similarly,

(14)H1=P(C0|S1)H(R10)+P(C1|S1)H(R11)(15)=P(C0|S1)H(R10)+[1−P(C0|S1)]H(R11),

which is also a function of the misclassification rate P(C0|S1).

The overall bitrate (BR) in theory can be obtained as follows by probabilistically combining the individual bitrates for the four cases. The individual bitrates can be represented by the entropies of the residues $H(R_{ij})$ since lossless compression is used.

(16) $$\begin{array}{c} BR=\sum_{i=0}^{1}\sum_{j=0}^{1}P\left(Si,Cj\right)H\left(R_{ij}\right). \end{array}$$

We can see that the overall bitrate can also be obtained by probabilistically combining the conditional entropies H0 and H1 in Equations (13) and (15) as follows:

(17) $$\begin{array}{c} BR=H0\cdot P(S0)+H1\cdot P(S1), \end{array}$$

which shows that the overall bitrate is a function of the misclassification rates.

In practice, the residue sources can be modeled by the geometric distributions with varying parameters $p_{ij}$ (corresponding to one of the four possible cases of image classifications $\left(Si,C_{j}\right)$). That is, the probability mass functions of the residue sources are

(18) $$\begin{array}{c} \mathrm{Prob}\left(n\right)=p_{ij}^{n}\left(1-p_{ij}\right), \end{array}$$

where *n* denotes the values of residues, and i,j=0,1. Therefore, we can use Equation (2) to replace $H(R_{ij})$ with the entropy of the geometric source:

(19) $$\begin{array}{c} H\left(R_{ij}\right)=\frac{-\left(1-p_{ij}\right)\cdot \log_{2}\left(1-p_{ij}\right)-p_{ij}\cdot \log_{2}p_{ij}}{1-p_{ij}}. \end{array}$$

Furthermore, we can derive the following formula for estimating the average codeword lengths (ACWL in bits, which is the practically achievable bitrates) over all four cases when we employ Golomb–Rice codes to compress the residues.

(20)ACWLOverall=∑i=01∑j=01P(Si,Cj)·ACWL(Rij)(21)=∑i=01∑j=01P(Cj|Si)·P(Si)·ACWL(Rij),

where $ACWL(R_{i,j})$ denotes the average codeword length of Golomb–Rice coding the residue source $R_{ij}$, which can be estimated by using Equation (7). We can see that the overall average codeword length is a function of the misclassification rates P(C1|S0) and P(C0|S1).

## 3. Results and Discussion

For the purpose of visualizing this relation revealed by the foregoing theoretic analysis, we simply assume that the cells are equally likely to be either normal or infected, i.e., $P\left(S0\right)=P\left(S1\right)=\frac{1}{2}$. Note here the theoretical results obtained in the previous section can handle other more general situations, e.g., the there will be more normal cells than infected cells, or the two misclassification rates are different. However, making the above simplifying assumptions can allow for 2D plotting of the relations between compression performance and a single misclassification rate.

We use two image datasets (with 400 images for each class) to estimate the compression performance. We first train two stacked autoencoders, one for normal cells and the other for infected cells. Then we vary the misclassification rates from 0.01 to 0.2 with a step size of 0.01. We then formulate the mixed images datasets according to the misclassification rates. For example, if the misclassification rate P(C1|S0)=P(C0|S1)=0.1, then we will feed an image dataset consisting of 360 normal cells and 40 infected cells to the stacked autoencoders trained to compress normal cell images. Similarly, another image dataset consisting of 360 infected cells and 40 normal cells will be fed to the other stacked autoencoders trained to compress infected cell images.

### 3.1. Conditional Entropies Versus Misclassification Rates

**Figure 7:**
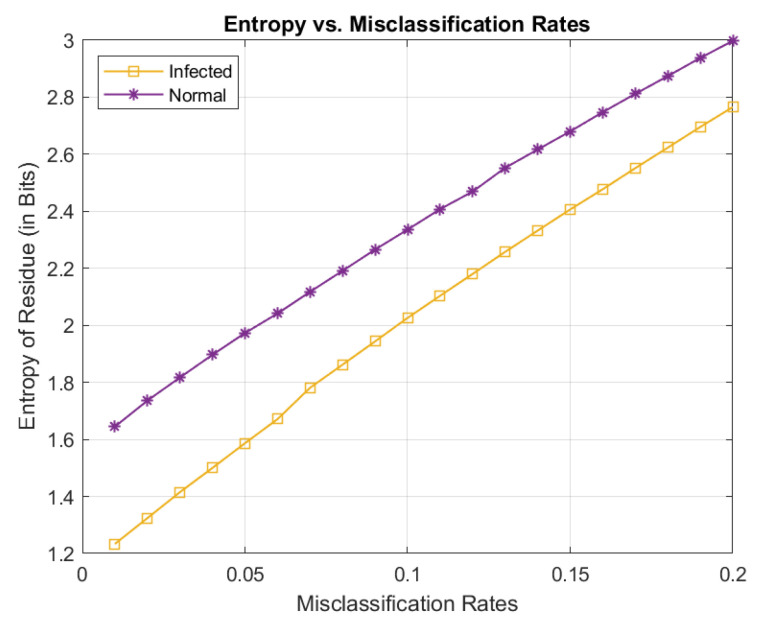
Estimated conditional entropies of the residues as a function of misclassification rates.

We first use Equations (13) and (15) to obtain the empirical entropies of the residues (conditional upon whether the inputs are normal or infected cells) as an estimate of the compressed bitrates. The results are plotted in Figure 7. We can see that the infected cells tend to be “easier” to compress than the normal cells. This can be attributed to the fact that infected cells share some common features, e.g., the existence of the ring form characteristic of parasite infection. While the autoencoders have been trained effectively capture the common features of the input images belonging to the same class, more and more “wrong” inputs from the other class due to misclassification lead to larger prediction residues, which translate to larger entropies, or lower compression. Thus for both classes of input images, we can see the apparent trend of lower and lower compression performance with an increasing misclassification rate, as expected.

### 3.2. Joint Entropy Versus Misclassification Rates

**Figure 8:**
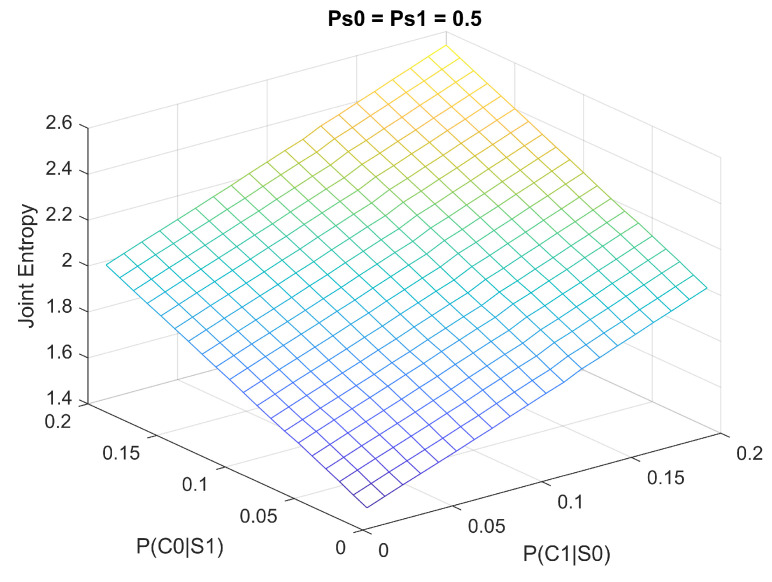
The joint entropy as a function of misclassification rates.

Here we still assume that the cells are equally likely to be either normal or infected, i.e., $P\left(S0\right)=P\left(S1\right)=\frac{1}{2}$, but allow the misclassification rates P(C1|S0), P(C0|S1) to change freely within the range. Based on Equation (16), we can plot a 3D surface as shown in Figure 8. We can see the general trend remains the same as the conditional entropies: when misclassification rates increase, the joint entropy (overall bitrates in theory) also increase.

### 3.3. Average Codeword Lengths Versus Misclassification Rates

**Figure 9:**
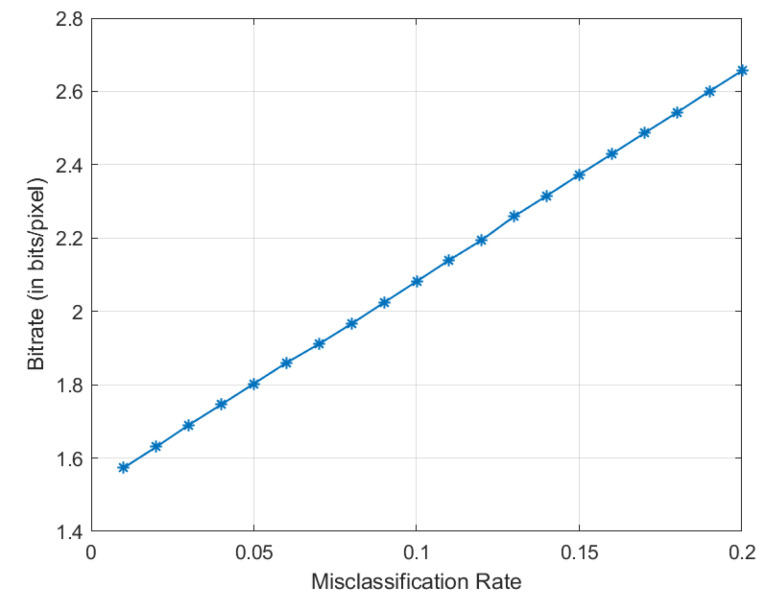
The overall average Golomb–Rice codeword lengths as a function of misclassification rates.

We use Golomb–Rice codes to compress the residues and use Equation (21) to calculate the average codeword lengths (ACWL in bits, which is the practically achievable bitrates) over all four cases (as shown in Figure 6). Figure 9 shows the relation between the overall ACWL (bitrates) and the misclassification rates. Again, the curve clearly shows the general trend of increased bitrates (less compression) when the misclassification rate increases, which is what we expected. In the following, we compare the compression performance of deep learning based method with some popular lossless image compression methods.

### 3.4. Comparisons with Mainstream Lossless Compression Methods

We compare with four well known lossless image compression methods. A brief introduction to these methods is given below.

- JPEG2000 [49] is an image compression standard designed to improve the performance of JPEG compression standard, albeit at the cost of increased computational complexity. Instead of using DCT in JPEG, JPEG2000 uses discrete wavelet transform (DWT).
- JPEG-LS is a lossless image compression standard. JPEG-LS improves the compression by using more context pixels (pixels already encoded) to predict the current pixel [50]. We use the codec based on the LOCO-I algorithm [51].
- CALIC (Context-based, adaptive, lossless image codec) uses a large number of contexts to condition a non-linear predictor, which makes it adaptive to varying source statistics [52].
- WebP [53] is an image format currently developed by Google. WebP is based on block prediction, and a variant of LZ77-Huffman coding is used for entropy coding.

**Figure 10:**
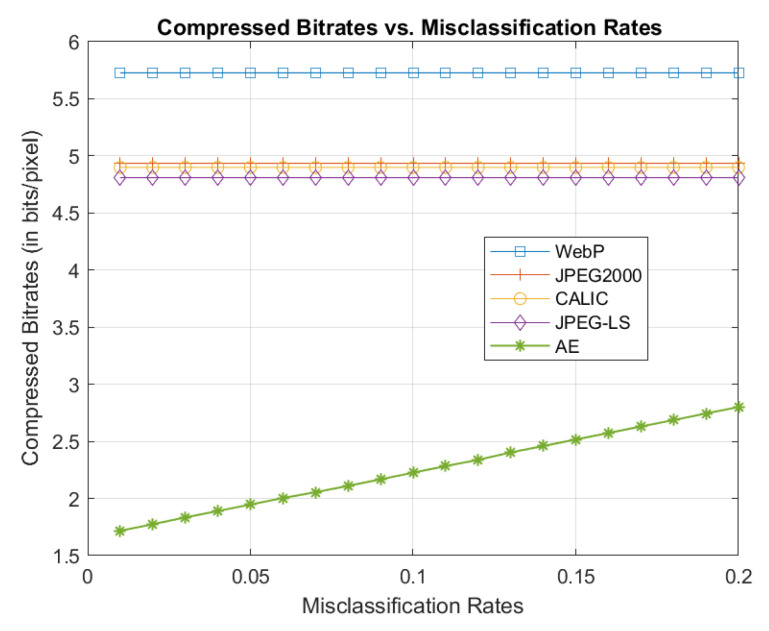
Comparison of bitrates for varying misclassification rates.

The comparison results are shown in Figure 10. We can see that our method significantly outperforms other four conventional compression methods, which are not sensitive to the change of the misclassification rates. This is because these standard methods are designed to be as generic as possible, without taking advantage of the correlations among images belonging to the same classes, which can be captured by sufficiently trained autoencoders. Here we take into account practical scenarios where there will be mismatch between the input images and the autoencoders of the corresponding class. For example, the autoencoders pre-trained to compress infected cell images would suffer from degrading performance as more and more normal cell images (due to increasing misclassification rates) are mixed with the infected cells as the input. However, even at a very low misclassification rate of 20% (which a reasonably good pattern classifier can easily do better in terms of accuracy), the curve Figure 10 shows the deep learning based method still has better performance than the four other methods.

The result highlights the advantage of our data-specific approach of “train once and then compress many times”, where deep learning seems to be very effective in extracting common features within the dataset, thereby providing more efficient data compression. Nonetheless, in practical implementations of an end-to-end compression/decompression system, the parameters of the stack autoencoders already trained have to be provided as side information to the decoder to ensure lossless decompression. Fortunately, this one-time cost of bitrates for the side information can be amortized over a large number of images to be compressed in the dataset. The other side information is the 30-point vector for each image at the output of the autoencoder at the last stage. Again, the bits needed for coding the vector is a one-time cost for the entire image, representing an negligible increase in the average bitrates (in bits/pixel).

It should also be noted that this deep learning based approach has some limitations. First, the approach is more suitable for achieving good compression on average over an entire dataset, where images can be grouped into different classes by a reasonably well trained classifier. The images within the same class share some common features, which can be exploited to achieve higher compression than would be possible by considering only individual image statistics. Therefore, this joint classification/compression approach is not intended for compression of individual images, for which mainstream lossless compression methods are more suitable, since they optimize their performance based on individual image statistics. Second, training stacked autoencoders on large dataset tend to be expensive computationally. Therefore, the high computational cost will only justify the “train once and then compress many times” approach applied on the entire dataset. Finally, the autoencoder parameters (e.g., the weights and biases of each layer) have to be made available to the decoder as a side information. Therefore, the advantage of the deep learning based method would be more pronounced for large datasets, where the impact of the side information overhead on the overall bitrates will become less noticeable for the entire dataset.

In the literature, existing work on deep learning for image compression is fairly sparse, mostly with the goal of achieving low bit rates and higher visual quality for lossy compression. For example, Toderici et al. proposed a general framework for variable-rate image compression based on convolutional and deconvolutional long short-term memory (LSTM) recurrent networks [54]. They reported better visual quality than JPEG2000 and WebP on 32×32 thumbnail images. Their follow-up work in [55] proposed a hybrid of Gated Recurrent Unit (GRU) and ResNet as a full-resolution lossy image compression methods. Jiang et al. [56] proposed an end-to-end lossy compression framework consisting of two convolutional neural networks (CNNs) for image compaction, albeit still requiring the main compression engine to be a standard compression method such as JPEG. Li et al. proposed a CNN-based content-weighted lossy compression method, which outperforms traditional methods on low bit rate images [57]. Generative Adversarial Networks (GANs) were used in [58] for lossy image compression, achieving good reconstructed image quality at very low bit rates (e.g., below 0.1 bit per pixel). In contrast, this work focuses on lossless compression. Our results shows that autoencoders are capable of capturing inter-image correlations in a large datasets, which are beneficial to efficient lossless compression of the entire dataset. It would be a good research direction to study how to integrate autoencoders with other deep learning architectures such as CNNs and GANs to exploit also local image statistics, as well as recurrent neural networks (RNNs) and LSTM networks to take advantage of pixel dependence within an image.

## 4. Conclusions

In this paper, we study how the performance of lossless compression on red blood cell images is affected by an imperfect classifier in a realistic setting where images are first classified prior to being compressed using deep learning methods based on stacked autoencoders. We provide an in-depth analysis on the impact of misclassification rates on the overall image compression performance and derive formulas for both empirical entropy and average codeword lengths based on Golomb–Rice codes for residues. These formulas provide new insight into how the overall compression efficiency are affected by different source probability and misclassification rates. We also use malaria infection image datasets to evaluate the relations between misclassification rates and actually obtainable compressed bit rates. The results show the advantage of our data driven approach of “train the neural network once and then compress the data many times”, where deep learning seems to be very effective in extracting common features within the dataset, thereby providing more efficient data compression than conventional methods, even at elevated misclassification rates. This special feature will be useful when only some important parts (regions of interest) of a large high-resolution (e.g., a wholeslide image) are required for lossless compression, while the rest (e.g., the background) only need lossy compression, or can simply be discarded. In the case of computer assisted malaria diagnosis, pathologists are mainly interested in red blood cell images. So we can classify the infected and normal cells, which can lead to more efficient compression of an entire image datasets. Thus, the proposed compression method can find useful applications in telemedicine where efficient storage and rapid transfer of large image datasets is sought after. As future work, we aim to study the compression performance and computational efficiencies of an end-to-end classification/compression system, taking into account the overhead associated with the descriptions of the neural network structure and feature vectors.

## Figures and Tables

**Table 1:** Meanings of the probabilities involved in the binary channel model.

Symbols	Meaning
P(S0)	Source probability of a normal cell image
P(S1)	Source probability of an infected cell image
P(C0|S0)	Conditional probability of a normal cell being correctly classified
P(C1|S0)	Cond. prob. of a normal cell being incorrectly classified as an infected cell
P(C0|S1)	Cond. prob. of an infected cell being incorrectly classified as a normal cell
P(C1|S1)	Cond. prob. of an infected cell being correctly classified
P(S0,C0)	Joint probability of a cell being normal and correctly classified
P(S0,C1)	Joint prob. of a cell being normal but incorrectly classified as an infected cell
P(S1,C0)	Joint prob. of a cell being infected but incorrectly classified as a normal cell
P(S1,C1)	Joint prob. of a cell being infected and correctly classified

## References

[1] Chan C. (2015). World Malaria Report.

[2] Kettelhut M.M., Chiodini P.L., Edwards H., Moody A. (2003). External quality assessment schemes raise standards: Evidence from the UKNEQAS parasitology subschemes. J. Clin. Pathol..

[3] Delahunt C.B., Mehanian C., Hu L., McGuire S.K., Champlin C.R., Horning M.P., Wilson B.K., Thompon C.M. Automated microscopy and machine learning for expert-level malaria field diagnosis. Proceedings of the 2015 IEEE Global Humanitarian Technology Conference (GHTC).

[4] Muralidharan V., Dong Y., Pan W.D. A comparison of feature selection methods for machine learning based automatic malarial cell recognition in wholeslide images. Proceedings of the 2016 IEEE-EMBS International Conference on Biomedical and Health Informatics (BHI).

[5] Park H.S., Rinehart M.T., Walzer K.A., Chi J.T.A., Wax A. (2016). Automated Detection of P. falciparum Using Machine Learning Algorithms with Quantitative Phase Images of Unstained Cells. PLoS ONE.

[6] Sanchez C.S. (2015). Deep Learning for Identifying Malaria Parasites in Images. Master’s Thesis.

[7] Quinn J.A., Nakasi R., Mugagga P.K.B., Byanyima P., Lubega W., Andama A. Deep Convolutional Neural Networks for Microscopy-Based Point of Care Diagnostics. Proceedings of the International Conference on Machine Learning for Health Care.

[8] Center for Devices and Radiological Health (2015). Technical Performance Assessment of Digital Pathology Whole Slide Imaging Devices.

[9] Farahani N., Parwani A.V., Pantanowitz L. (2015). Whole slide imaging in pathology: Advantages, limitations, and emerging perspectives. Pathol. Lab. Med. Int..

[10] University of Alabama at Birmingham PEIR-VM. http://peir-vm.path.uab.edu/about.php.

[11] Cornish T.C. An Introduction to Digital Wholeslide Imaging and Wholeslide Image Analysis. https://docplayer.net/22756037-An-introduction-to-digital-whole-slide-imaging-and-whole-slide-image-analysis.html.

[12] Al-Janabii S., Huisman A., Nap M., Clarijs R., van Diest P.J. (2012). Whole Slide Images as a Platform for Initial Diagnostics in Histopathology in a Medium-sized Routine Laboratory. J. Clin. Pathol..

[13] Pantanowitz L., Valenstein P., Evans A., Kaplan K., Pfeifer J., Wilbur D., Collins L., Colgan T. (2011). Review of the current state of whole slide imaging in pathology. J. Pathol. Inform..

[14] Tek F.B., Dempster A.G., Kale I. (2009). Computer vision for microscopy diagnosis of malaria. Malar. J..

[15] World Health Organization Microscopy. http://www.who.int/malaria/areas/diagnosis/microscopy/en/.

[16] Halim S., Bretschneider T.R., Li Y., Preiser P.R., Kuss C. Estimating malaria parasitaemia from blood smear images. Proceedings of the IEEE International Conference on Control, Automation, Robotics and Vision.

[17] Das D., Ghosh M., Chakraborty C., Pal M., Maity A.K. Invariant Moment based feature analysis for abnormal erythrocyte segmentation. Proceedings of the International Conference on Systems in Medicine and Biology (ICSMB).

[18] Das D.K., Ghosh M., Pal M., Maiti A.K., Chakraborty C. (2013). Machine learning approach for automated screening of malaria parasite using light microscopic images. J. Micron.

[19] Tek F.B., Dempster A.G., Kale I. (2010). Parasite detection and identification for automated thin blood film malaria diagnosis. J. Comput. Vis. Image Underst..

[20] Di Ruberto C., Dempster A., Khan S., Jarra B. (2002). Analysis of infected blood cell images using morphological operators. J. Comput. Vis. Image Underst..

[21] Ross N.E., Pritchard C.J., Rubin D.M., Duse A.G. (2005). Automated image processing method for the diagnosis and classification of malaria on thin blood smears. Med. Biol. Eng. Comput..

[22] Makkapati V.V., Rao R.M. Segmentation of malaria parasites in peripheral blood smear images. Proceedings of the IEEE International Conference on Acoustics, Speech, and Signal Processing.

[23] Tek F.B., Dempster A.G., Kale I. Malaria parasite detection in peripheral blood images. Proceedings of the British Machine Vision Conference 2006.

[24] Linder N., Turkki R., Walliander M., Mårtensson A., Diwan V., Rahtu E., Pietikäinen M., Lundin M., Lundin J. (2014). A Malaria Diagnostic Tool Based on Computer Vision Screening and Visualization of Plasmodium falciparum Candidate Areas in Digitized Blood Smears. PLoS ONE.

[25] Pan W.D., Dong Y., Wu D., Farhadi H. (2018). Classification of Malaria-Infected Cells Using Deep Convolutional Neural Networks. Machine Learning—Advanced Techniques and Emerging Applications.

[26] Shen H., Pan W.D., Dong Y., Alim M. Lossless compression of curated erythrocyte images using deep autoencoders for malaria infection diagnosis. Proceedings of the IEEE Picture Coding Symposium (PCS).

[27] Duh D.J., Jeng J.H., Chen S.Y. (2005). DCT based simple classification scheme for fractal image compression. Image Vis. Comput..

[28] Fahmy G., Panchanathan S. A lifting based system for optimal compression and classification in the JPEG2000 framework. Proceedings of the IEEE International Symposium on Circuits and Systems (ISCAS 2002).

[29] Kim H., Yazicioglu R.F., Merken P., Van Hoof C., Yoo H.J. (2010). ECG signal compression and classification algorithm with quad level vector for ECG holter system. IEEE Trans. Inf. Technol. Biomed..

[30] Jha C.K., Kolekar M.H. (2018). Classification and Compression of ECG Signal for Holter Device. Biomedical Signal and Image Processing in Patient Care.

[31] Minguillón J., Pujol J., Serra J., Ortimo I. (2000). Influence of lossy compression on hyperspectral image classification accuracy. WIT Trans. Inf. Commun. Technol..

[32] Garcia-Vilchez F., Muñoz-Marí J., Zortea M., Blanes I., González-Ruiz V., Camps-Valls G., Plaza A., Serra-Sagristà J. (2011). On the impact of lossy compression on hyperspectral image classification and unmixing. IEEE Geosci. Remote Sens. Lett..

[33] Gelli G., Poggi G. (1999). Compression of multispectral images by spectral classification and transform coding. IEEE Trans. Image Process..

[34] Peng K., Kieffer J.C. (2004). Embedded image compression based on wavelet pixel classification and sorting. IEEE Trans. Image Process..

[35] Oehler K.L., Gray R.M. Combining image classification and image compression using vector quantization. Proceedings of the IEEE Data Compression Conference (DCC’93).

[36] Oehler K.L., Gray R.M. (1995). Combining image compression and classification using vector quantization. IEEE Trans. Pattern Anal. Mach. Intell..

[37] Li J., Gray R.M., Olshen R. Joint image compression and classification with vector quantization and a two dimensional hidden Markov model. Proceedings of the Data Compression Conference, DCC’99.

[38] Baras J.S., Dey S. (1999). Combined compression and classification with learning vector quantization. IEEE Trans. Inf. Theory.

[39] Ayoobkhan M.U.A., Chikkannan E., Ramakrishnan K., Balasubramanian S.B. Prediction-Based Lossless Image Compression. Proceedings of the International Conference on ISMAC in Computational Vision and Bio-Engineering 2018 (ISMAC-CVB).

[40] Fu D., Guimaraes G. Using Compression to Speed Up Image Classification in Artificial Neural Networks. http://www.danfu.org/files/CompressionImageClassification.pdf.

[41] Andono P.N., Supriyanto C., Nugroho S. (2018). Image compression based on SVD for BoVW model in fingerprint classification. J. Intell. Fuzzy Syst..

[42] Mohanty I., Pattanaik P.A., Swarnkar T. Automatic Detection of Malaria Parasites Using Unsupervised Techniques. Proceedings of the International Conference on ISMAC in Computational Vision and Bio-Engineering 2018 (ISMAC-CVB).

[43] Whole Slide Image Data. http://peir-vm.path.uab.edu/debug.php?slide=IPLab11Malaria.

[44] Dong Y., Jiang Z., Shen H., Pan W.D., Williams L.A., Reddy V.V., Benjamin W.H., Bryan A.W. Evaluations of deep convolutional neural networks for automatic identification of malaria infected cells. Proceedings of the 2017 IEEE EMBS International Conference on Biomedical & Health Informatics (BHI).

[45] Duda R.O., Hart P.E. (1972). Use of the Hough transformation to detect lines and curves in pictures. Commun. ACM.

[46] Link to the Dataset Used. http://www.ece.uah.edu/~dwpan/malaria_dataset/.

[47] Hinton G.E., Salakhutdinov R.R. (2006). Reducing the dimensionality of data with neural networks. Science.

[48] Golomb S. (1966). Run-length encodings (Corresp.). IEEE Trans. Inf. Theory.

[49] JPEG2000 Home Page. https://jpeg.org/jpeg2000/.

[50] JPEG-LS Home Page. https://jpeg.org/jpegls/.

[51] Weinberger M.J., Seroussi G., Sapiro G. (2000). The LOCO-I lossless image compression algorithm: Principles and standardization into JPEG-LS. IEEE Trans. Image Process..

[52] Wu X., Memon N. CALIC—A context based adaptive lossless image codec. Proceedings of the 1996 IEEE International Conference on Acoustics, Speech, and Signal Processing (ICASSP-96).

[53] WebP Home Page. https://developers.google.com/speed/webp/.

[54] Toderici G., O’Malley S.M., Hwang S.J., Vincent D., Minnen D., Baluja S., Covell M., Sukthankar R. (2015). Variable Rate Image Compression with Recurrent Neural Networks. arXiv.

[55] Toderici G., Vincent D., Johnston N., Hwang S., Minnen D., Shor J., Covell M. Full Resolution Image Compression with Recurrent Neural Networks. Proceedings of the IEEE Conference on Computer Vision and Pattern Recognition (CVPR).

[56] Jiang F., Tao W., Liu S., Ren J., Guo X., Zhao D. (2018). An End-to-End Compression Framework Based on Convolutional Neural Networks. IEEE Trans. Circuits Syst. Video Technol..

[57] Li M., Zuo W., Gu S., Zhao D., Zhang D. Learning Convolutional Networks for Content-Weighted Image Compression. Proceedings of the 2018 IEEE/CVF Conference on Computer Vision and Pattern Recognition.

[58] Agustsson E., Tschannen M., Mentzer F., Timofte R., Van Gool L. Generative Adversarial Networks for Extreme Learned Image Compression. Proceedings of the IEEE Conference on Computer Vision and Pattern Recognition (CVPR) Workshops.

